# The role of metallothionein in a dinitrofluorobenzene-induced atopic dermatitis-like murine model

**DOI:** 10.1038/s41598-018-29410-w

**Published:** 2018-07-24

**Authors:** Jin-Zhu Guo, Wen-Hui Wang, Lin-Feng Li, Shao-Min Yang, Jing Wang

**Affiliations:** 1 Peking University Third Hospital, Department of Dermatology, Beijing, 100191 China; 2 Capital Medical University Affiliated Beijing Friendship Hospital, Department of Dermatology, Beijing, 100050 China; 3 Peking University Health Science Center, Department of Pathology, Beijing, 100191 China

## Abstract

Atopic dermatitis (AD), one of the most common chronic eczematous skin disorders, is associated with cutaneous hyperactivity as a response to environmental triggers. Metallothionein (MT) plays a constitutive defensive role in the response to noxious stimuli. However, the role of MT in AD development is unclear. Using an AD-like murine model created by the topical application of 2.4-dinitrofluorobenzene, we studied the dynamic pattern of MT expression on AD development. AD-like lesions were evaluated based on the development of erythema, edema, exfoliation, scaling, increased thickness, and increased weight of lesional skin. These characteristics of AD-like lesions and thymic stromal lymphopoietin (TSLP) expression peaked at Day 1 of the establishment of our model and gradually alleviated over time. The MT expression in lesional skin was increased and peaked at Day 3. By immunostaining, increased expression of MT was translocated from the cytoplasm to the nucleus. MT-1/2 knockout (*MT*−/−) mice and wild type (*MT*+/+) mice were also used to evaluate the role of MT on AD. *MT*−/− mice had greater edema scores, thickness, lesional skin weight, as well as more CD4+ T cells, TSLP, superoxide dismutase, and NDUFAF1. These results suggest that MT may play a protective role against AD development, and that antioxidant and nuclear protective mechanisms may be involved.

## Introduction

Metallothioneins (MTs) are a group of endogenous, soluble, low-molecular-weight (7 kDa), heat-stable and metal-binding proteins found in mammals. They each contain about 60 amino acids. In mice, there are four MT genes that reside in a 50 kb region on chromosome 8. In humans, the MT proteins are encoded by a family of genes located on chromosome 16q13, which includes at least ten identified functional genes^1–3^. MT is classified into major and minor groups. The major group includes *MT-1* and *MT-2*, which are both expressed universally. In contrast, MT-3 and MT-4 belong to the minor isoforms, which are normally found in specialized cells^1,2^.

The *MT-1* and *MT-2* genes are expressed in skin. They participate in cell transcription, detoxify heavy metals, modulate immune function, and participate in a variety of gastrointestinal tract functions. The expression of MT-1/2 is increased when exposed to metals including Hg, Cd, Cu, and Zn, in addition to cytokines and reactive oxygen species (ROS)^1–3^. *MT-1/2* null (*MT*−/−) mice exhibited reduced tolerance to ultraviolet B injury^3^, sodium lauryl sulphate (SLS)-induced skin irritation^4^, and ovalbumin-related airway inflammation^5^, suggesting that MTs play a protective role against these diseases.

Atopic dermatitis (AD) is a chronic recurrent inflammatory skin disease. Its complex etiology includes abnormal immunological and inflammatory responses that lead to skin barrier disruption, which result in the loss of proper protection when exposed to environmental agents and neuropsychological factors. A number of murine AD models have been developed, including models induced by the epicutaneous application of sensitizers, transgenic mice models, and models formed from mice that spontaneously develop AD-like skin lesions^6–14^. Haptens, such as oxazolone, trinitrochlorobenzene (TNCB) and dinitrofluorobenzene (DNFB), are commonly used to induce murine models of contact hypersensitivity. It has been reported that the repeated application of these haptens over an extended period causes skin inflammation to shift from a typical Th1-dominated contact hypersensitivity response to a Th2-dominated inflammatory response that is similar to human AD^15,16^. Based on this observation, hapten-induced models have been used for evaluating pathogenic mechanisms and potential therapies of AD^6–14^. A DNFB-induced Th2-dominated AD-like murine model was successfully used in our previous study^17,18^. In that model, Th2 type inflammation markers, such as IgE, IL-4, IL-17A, and the number of CD4+ T cell and mast cells, were found to be increased significantly, whereas Th1 type markers, such as IL-2, IFN-γ, IL-1β, and TNF-α, were significantly decreased^17,18^. Using a DNFB-induced AD-like murine model, we studied the role MTs play in AD development and their potential mechanisms.

## Results

### MT expression pattern in DNFB-induced AD-like lesion in BALB/c mice

**Figure 1:**
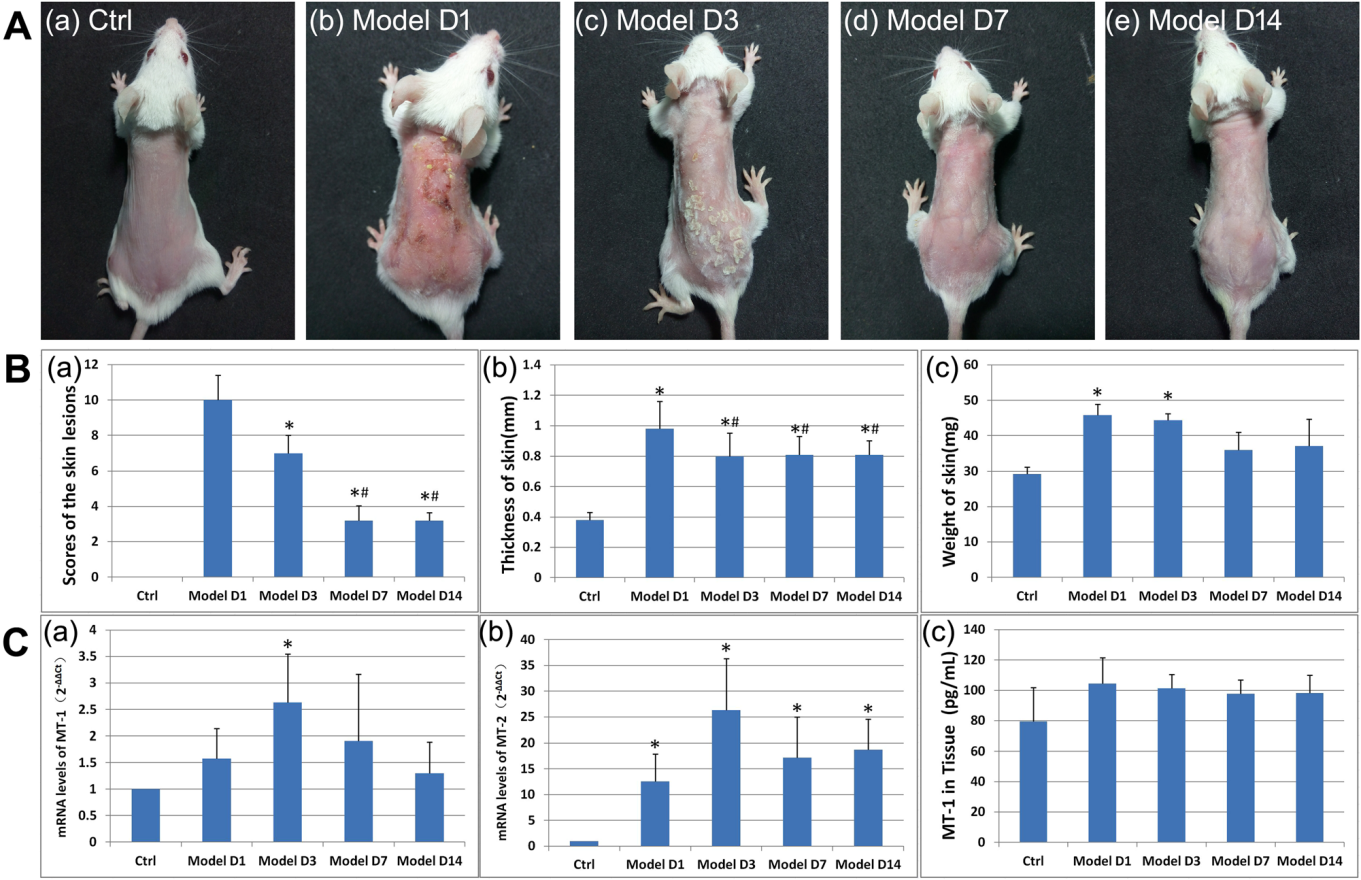
DNFB-induced AD-like lesions in BALB/c mice and MT dynamics. (**A**) Clinical picture of DNFB-induced AD-like lesions. (**B**) Evaluation of DNFB-induced AD-like lesions. (a) Scores of the lesions. Skin lesions of erythema, edema, exfoliation, and scaling were scored as 0 (none), 1 (mild), 2 (moderate), and 3 (severe)^17,18^. *P < 0.01 versus model group on Day 1. ^#^P < 0.01 versus model group on Day 3. (b) The thickness of the skin. Skin thickness was measured three times with three different sites of the back. The average values were presented. *P < 0.01 versus control group. ^#^P < 0.01 versus model group on Day 1. (c) The weight of the skin. Dorsal skin measuring 6 mm × 6 mm at a fixed position across all the mice was sheared without subcutaneous fat. *P < 0.001 versus control group. (**C**) (a,b) The expression of the mRNA level of *MT-1* and *MT-2* in the skin tissue. The primary value of Ct was transferred into 2^−ΔΔCt^. *P < 0.01 versus control group. (c) The expression of the protein level of *MT-1* in skin tissue.

The erythema, edema, exfoliation, and scaling scores of DNFB-induced AD-like lesions in BALB/c mice (Fig. 1A) reached maximum values at Day 1 after the model was created, and the scores were significantly higher than in other groups (P < 0.01) (Fig. 1B(a)). The score at Day 3 was still significantly higher than that at Day 7 and Day 14 (P < 0.01) (Fig. 1B(a)).

Skin thickness increased significantly in model groups compared to the control group (P < 0.01). The thickness of the lesions at Day 1 was significantly greater than that at Day 3, Day 7, and Day 14 (P < 0.01). No significant differences were found between Day 3, Day 7, and Day 14 (P > 0.01) (Fig. 1B(b)).

The weight of lesional skin increased significantly in the model groups at Day 1 and Day 3 compared to the control group (P < 0.001). There were no significant differences between Day 7, Day 14, and the control group (P > 0.01) (Fig. 1B(c)).

The *MT-1* mRNA level increased significantly at Day 3 compared to that in the control group (P < 0.01) (Fig. 1C(a)). Although no significant differences were found, the levels of *MT-1* mRNA at Day 1, Day 7, and Day 14 were higher than those in the control group. The levels of *MT-2* mRNA were significantly higher in all the model groups than in the control group (P < 0.01), with the maximum being reached at Day 3 (Fig. 1C(b)).

The levels of *MT-1* protein in the skin tissue were higher in all the model groups than in the control group, although the differences were not significant (Fig. 1C(c)).

**Figure 2:**
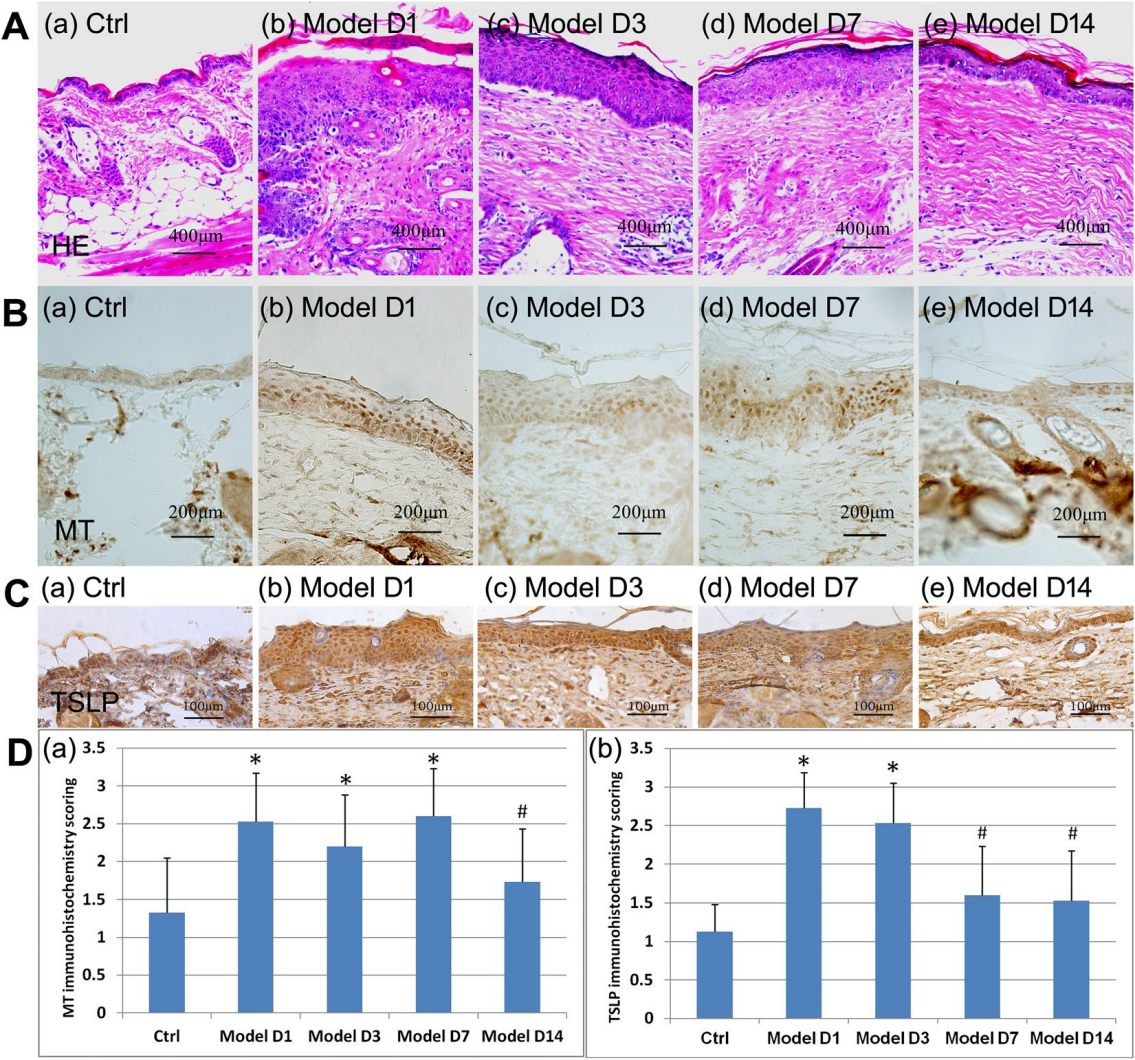
Immunohistology of MT and TSLP in DNFB-induced AD-like lesions in BALB/c mice. (**A**) Histopathological (H&E). (**B**) Immunostaining of MT. (**C**) Immunostaining of TSLP. (**D**) Immunohistochemical scoring of MT and TSLP. (a) Immunohistochemical scoring of MT in BALB/c mice. *P < 0.01 versus control group. ^#^P < 0.01 versus model group on Day 1 and Day 7. (b) Immunohistochemical scoring of TSLP in BALB/c mice. *P < 0.01 versus control group. ^#^P < 0.01 versus model group on Day 1 and Day 3.

Histopathology of the skin was examined by H&E (Fig. 2A(a–e)). In the control group, MT was weakly stained in the cytoplasm and relatively homogeneously distributed (Fig. 2B(a)). The model groups exhibited strongly-stained nuclei with lighter or similar cytoplasm staining of MT (Fig. 2B(b–e)), particularly within the lower part of epidermis, compared to the control group. Staining scores of MT were significantly higher in the model groups at Day 1, Day 3, and Day 7 than in the control group (P < 0.01) and decreased to a level like the control group by Day 14 (P > 0.01) (Fig. 2D(a)).

Some basal level of thymic stromal lymphopoietin (TSLP) was detected in the skin of the control group (Fig. 2C(a)). In contrast, numerous cells, particularly keratinocytes, were stained positive by anti-TSLP in the skin of the model group, particularly at Day 1 and Day 3 (Fig. 2C(b–e)). The staining scores of TSLP were significantly higher in the model groups at Day 1 and Day 3 than in the control group (P < 0.01) and decreased to a level like the control group by Day 7. (P > 0.01) (Fig. 2D(b)).

### The difference between the AD-like models of MT−/− and MT+/+ mice

**Figure 3:**
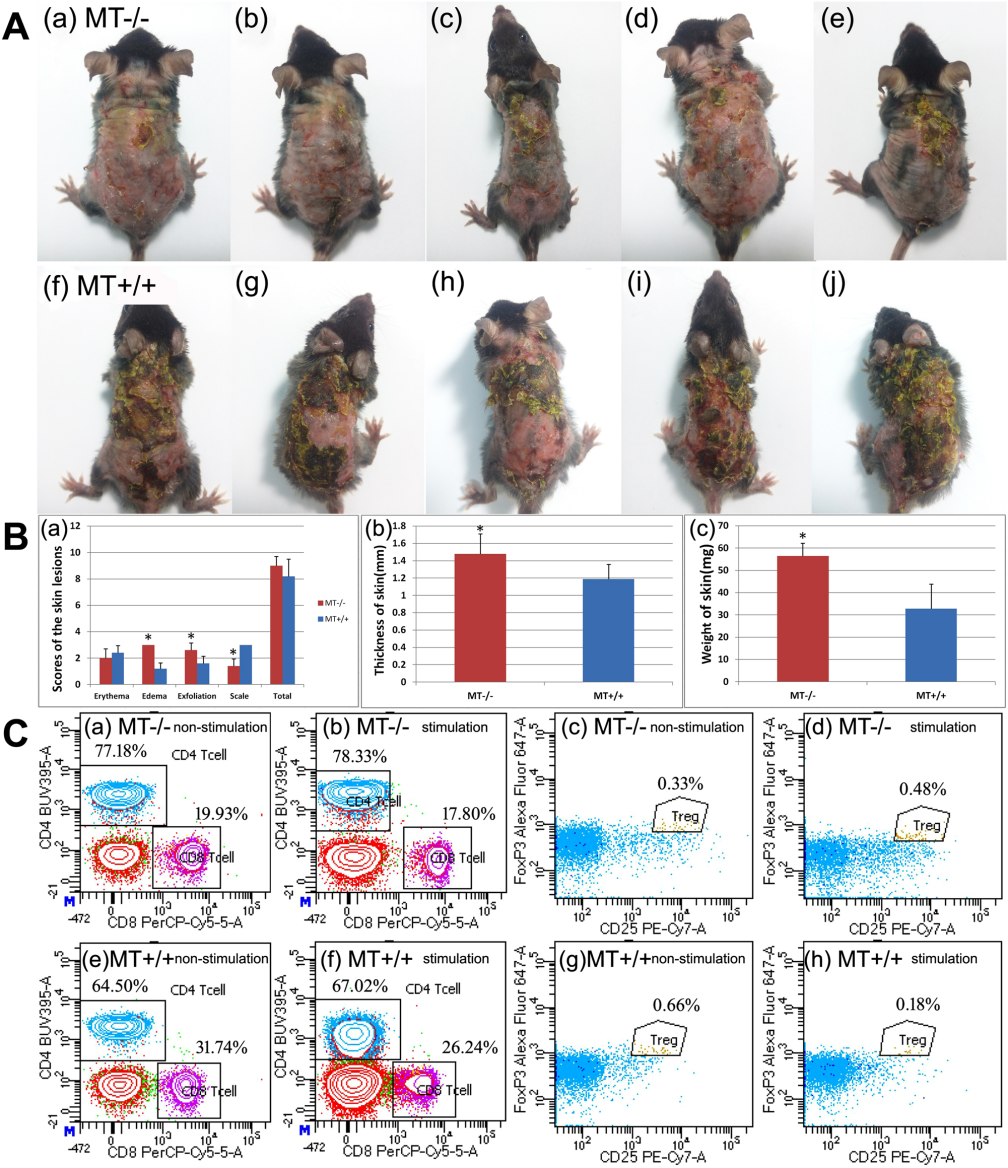
Comparisons of DNFB-induced AD-like lesion and FACS analyses between MT−/− and MT+/+ mice. (**A**) Clinical picture of DNFB-induced AD-like lesions on Day 1 after model establishment. (a–e) *MT*−/− mice; (f–j) *MT*+/+ mice. (**B**) Evaluation of DNFB-induced AD-like lesions in *MT*−/− and *MT*+/+ mice. (a) Scores of the lesions. The erythema, edema, exfoliation, and scaling of lesional skin were scored as 0 (none), 1 (mild), 2 (moderate), and 3 (severe). (b) The thickness of the skin. This was randomly measured three times at three different sites of the back. The average values were analyzed and presented. (c) The weight of the skin. Dorsal skin measuring 6 mm × 6 mm on a fixed position in all the mice was sheared without subcutaneous fat. *P < 0.05 *MT*−/− versus *MT*+/+. (**C**) FACS analyses of CD4, CD8, and CD4+ CD25+ FoxP3+ Treg. (a–d) *MT*−/−: (a) CD4 and CD8 without PMA and ionomycin stimulation; (b) CD4 and CD8 with stimulation; (c) CD4+ CD25+ FoxP3+ Treg without stimulation; (d) CD4+ CD25+ FoxP3+ Treg with stimulation. (e-h) *MT*+/+: (e) CD4 and CD8 without PMA and ionomycin stimulation; (f) CD4 and CD8 with stimulation; (g) CD4+ CD25+ FoxP3+ Treg without stimulation; (h) CD4+ CD25+ FoxP3+ Treg with stimulation.

DNFB-induced AD-like lesions were successfully induced in both *MT*−/− and *MT*+/+ groups (Fig. 3A). In the *MT*−/− group, the edema and exfoliation scores were significantly higher, whereas the scaling score was significantly lower (P < 0.05). No significant differences were found in the erythema score and total scores between the *MT*−/− and *MT*+/+ groups (P > 0.05) (Fig. 3B(a)). The thickness and weight of skin of the *MT*−/− group were also significantly higher (P < 0.05) (Fig. 3B(b,c)).

Flow cytometric analysis of mononuclear cells in peripheral blood revealed that CD4/CD3 were significantly higher in the *MT*−/− mice regardless of treatment with (78.33% vs. 67.02%) (Fig. 3C(b,f)) or without (77.18% vs. 64.50%) (Fig. 3C(a,e)) PMA and ionomycin (P < 0.05). In contrast, CD8/CD3 in *MT*−/− were significantly lower regardless of treatment with (17.80% vs. 26.24%) (Fig. 3C(b,f)) or without (19.93% vs. 31.74%) (Fig. 3C(a,e)) both PMA and ionomycin (P < 0.05). CD4+/CD25+/FoxP3+ Treg cells in *MT*−/− was significantly higher when stimulated with PMA and ionomycin (0.48% vs. 0.18%) (Fig. 3C(d,h)) (P < 0.05). However, CD4+/CD25+/FoxP3+ Treg cells demonstrated no significant differences between the *MT*−/− and *MT*+/+ groups without PMA and ionomycin stimulation (0.33% vs. 0.66%) (Fig. 3C(c,g)) (P > 0.05). No differences in IL-4, IFN-γ, or IL-17a were found between the *MT*−/− and *MT*+/+ mice (P > 0.05).

**Figure 4:**
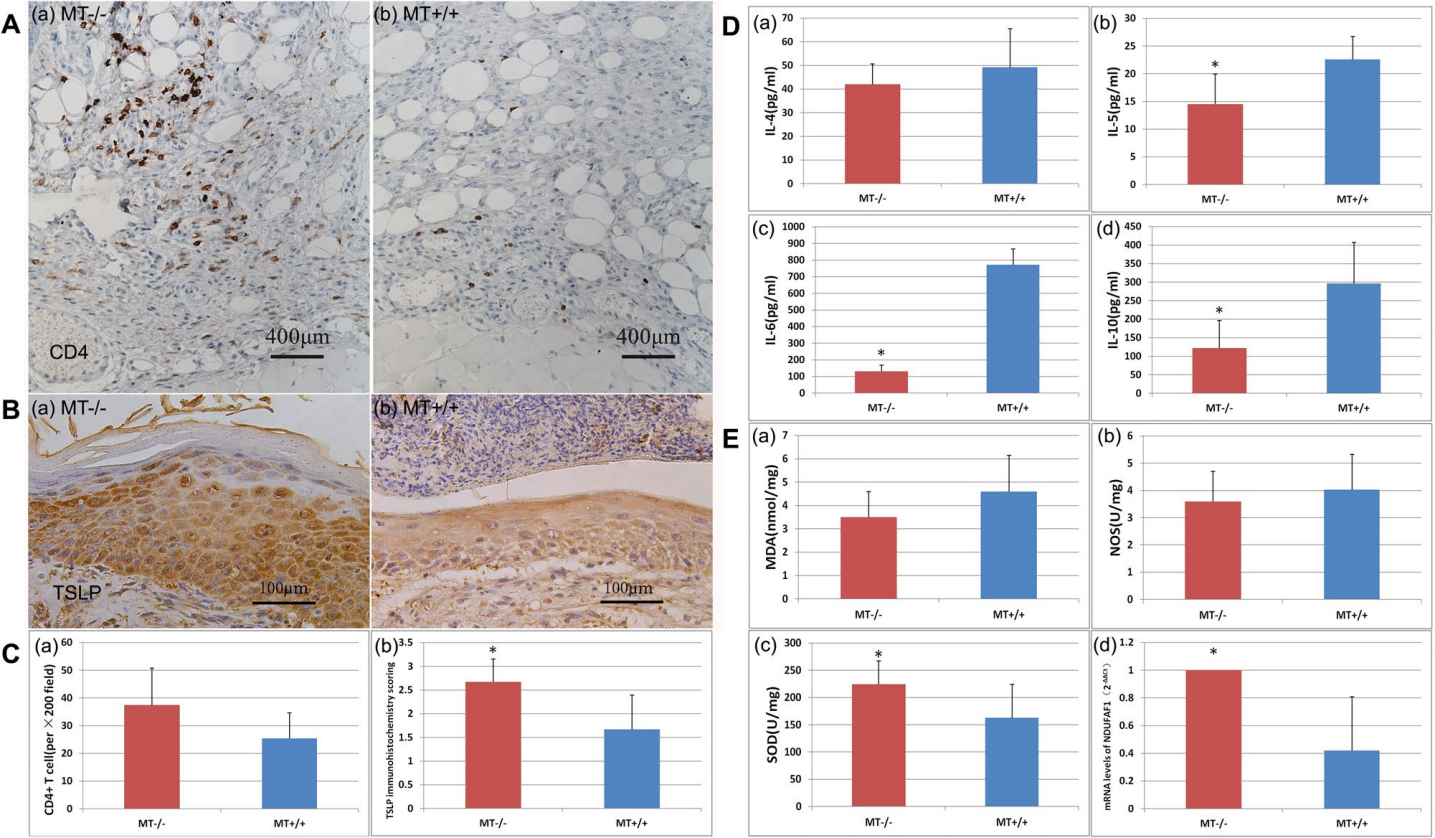
Comparison of Th2 cytokines and oxidative stress parameters in DNFB-induced AD-like lesions between *MT*−/− and *MT*+/+ mice. (**A**) Immunohistochemistry of CD4. (**B**) Immunohistochemistry of TSLP. (**C**) (a) CD4+ T cell number measured by counting five different areas in each slide (×200). (b) Immunohistochemical scoring of TSLP. (**D**) Comparisons of Th2 cytokines: IL-4, IL-5, IL-6, and IL-10. (**E**) Comparisons of oxidative stress parameters: MDA, NOS, SOD, and NDUFAF1. *P < 0.05 *MT*−/− versus *MT*+/+.

By immunohistochemistry staining, the average CD4+ T cell counts (five different continuous areas (×200)) were significantly higher in the *MT*−/− mice (P < 0.05) (Fig. 4A,C(a)).

Significantly increased levels of TSLP in immunohistochemistry were found in the skin of *MT*−/− compared with the *MT*+/+ groups (Fig. 4B,C(b)).

In the skin tissue homogenate supernatant, IL-5, IL-6, and IL-10 were significantly lower (P < 0.05) (Fig. 4D(b–d)) in the *MT*−/− mice. No significant differences in IL-4, malondialdehyde (MDA), and nitric oxide synthases (NOS) were found between the *MT*−/− and *MT*+/+ groups (P > 0.05) (Fig. 4D(a) and E(a,b)). Superoxide dismutase (SOD) was significantly higher (P < 0.05) (Fig. 4E(c)).

In addition, NDUFAF1 mRNA was significantly higher in the *MT*−/− than *MT*+/+ group (P < 0.05) (Mann-Whitney U test) (Fig. 4E(d)).

## Discussion

In this study, we reported that acute increased MT expression was correlated with the development of AD in an AD-like murine model, and mice without MT (*MT*−/−) developed more severe AD in comparison to wild type (*MT*+/+) mice. In response to topical contact of DNFB stimulation, increased expression of MT was translocated from the cytoplasm to the nucleus where MT might function and protect the nucleus from DNFB-induced damage. In *MT*−/− AD-like mice, a greater amount of CD4+ T cells and the expression of superoxide dismutase (SOD), an antioxidant enzyme, were detected, suggesting that MT is involved in the immune response and increased SOD might be a compensatory mechanism when MT is deficient. In addition, Th2 cell cytokines, including IL-5, IL-6, and IL-10, were significantly decreased in the MT-deficient AD-like mice, indicating that Th2-dominated inflammation was reduced in a MT-deficient environment.

Depending on the disease phase of AD (acute or subacute/chronic), the cytokine milieu is dominated by either Th2 cell cytokines with high levels of IL-4, IL-5, and IL-13, or Th1/0 cell cytokines including IFN-γ, IL-12, and GM-CSF^19^. In a mouse model in which Th1 or Th2 cells were injected into skin to induce a local inflammatory reaction, Th2 cells induced a rapid but short-lasting inflammation, whereas Th1 cells induced a later-onset but prolonged reaction^20^. These observations suggest that the initiation of AD was driven by Th2-type cell activation, whereas the chronic inflammatory reaction was driven by a Th1-type response. In our DNFB-induced AD-like murine model, Th2 cytokines were dominant^17,18^, supporting an acute AD-like lesion of activation phase. Our AD-like lesion exhibited the most severe response at Day 1 before being alleviated. The mRNA expression of *MT-1* and *MT-2* were increased at Day 1 and peaked at Day 3, suggesting that MT production was a reaction to inflammation and might be protective.

In the AD-like mouse model, enhanced total MT translocated from the cytoplasm to the nucleus. Overexpression of MT has been reported in patch test skin sites in humans, suggesting a defensive response following a chemical challenge^21^. A closer look at our model shows that the increased expression of MT was primarily concentrated in the nucleus. The nuclear localization of MT is associated with enhanced protection against oxidative stress and genomic damage, along with genomic regulation of other DNA-related proteins^22^. The high level of MT in the nucleus of cells under certain conditions might also be related to the need for higher levels of zinc for several metallo-enzymes and transcription factors during rapid growth. Consequently, the function of nuclear MT could be to protect the cell from DNA damage and cell apoptosis, as well as to regulate gene expression during certain stages of the cell cycle^23^.

To further verify the protective role of MT, additional experiments using MT-null mice were performed. In DNFB-induced AD-like lesions of *MT*−/− and *MT*+/+ mice, the edema and exfoliation score, skin thickness, and skin weight were all significantly higher in the *MT*−/− group. These results also support the conclusion that MT plays a protective role against AD development.

TSLP is released in the lesional epidermis in AD and acts as a master switch that triggers the Th2 and Th22 response^24^. The large amounts of TSLP in our model groups supported the successful establishment of the AD-like model by topical application of DNFB. The *MT*−/− group expressed stronger TSLP, further supporting the possible protective role of MT against AD development.

Significantly increased levels of CD4+ T cells and reduced levels of Th2 cell cytokines, including IL-5, IL-6, and IL-10, but not IL-4, were found in the MT−/− AD-like mice (Fig. 4A,D). Rice *et al*. found that MT regulated intracellular zinc signaling during CD4+ T cell activation. By expressing MT in response to certain environmental conditions, CD4+ T cells are able to release intracellular zinc and regulate signaling pathways more efficiently following stimulation^25^. These findings suggest that MT deficiency could influence T cell polarization in AD-like lesion.

MT modulates the exchange/transport of heavy metals such as zinc, cadmium, and copper under physiological conditions, exerts cytoprotection from heavy metal toxicities, and assists in the release of gaseous mediators such as hydroxyl radicals or nitric oxide^1^. MT, SOD, catalase, and glutathione peroxidase are antioxidant enzymes involved in cellular defense against reactive oxygen species (ROS). MT scavenges a wide range of ROS, including superoxide, hydrogen peroxide, hydroxyl radical, and nitric oxide (NO)^26^. In addition, lipids are the most susceptible to oxidation, and oxidative degradation of lipids produces malondialdehyde (MDA) and 4-hydroxynonenal^27^. In the current study, the higher level of SOD found in *MT*−/− AD-like mice could be a compensatory mechanism, whereas the antioxidant effect could not be fully compensated due to the lack of significant difference found between MDA and NOS.

NDUFAF1 is an essential chaperone for complex I assembly^28^. Kaminski *et al*. demonstrated that TCR-triggered ROS generation by complex I was indispensable for activation-induced IL-2 and IL-4 expression and secretion in resting and preactivated human T cells. IL-2 and IL-4 expression requires the synergistic action of the Ca^2+^ signal and the mitochondrial complex I-derived oxidative signal in the form of H_2_O_2_. By disrupting complex I or NDUFAF1, Kaminski and colleagues found that TCR-triggered ROS generation by complex I determined the activation of IL-2/-4 expression and secretion in resting and preactivated human T cells^28^. They also showed that the inhibition of mitochondrial complex I-mediated ROS generation led to a significant decrease in spontaneous hyperexpression and TCR-induced expression of IL-4 in T cells isolated from AD patients^28^. We found that NDUFAF1 mRNA was significantly elevated in *MT*−/− AD-like mice, indicating *MT*−/− group may generate more ROS by complex I.

In summary, MT may play a protective role, at least partially, in a DNFB-induced AD-like murine model. Antioxidant and nuclear protective mechanisms might be involved. Whether topical application of MT could prevent AD-like lesions and be used for treatment is a subject worthy of further study.

## Materials and Methods

### Animals

BALB/c mice, MT gene knockout mice (*MT*−/−) and homozygous wild-type mice with MT (*MT*+/+) were used. *MT*−/− and *MT*+/+ mice were developed by the Royal Children’s Hospital (Parkville, Melbourne, Australia). The mutations were introduced into embryonic stem cells by homologous recombination. Chimeric mice resulting from the targeted embryonic stem cells transmitted the disrupted alleles through their germ line. Homozygous animals were born alive and appeared phenotypically normal and fertile. These mice provide a useful model to study the physiological roles of *MT-I* and *MT-II*^29^. All experimental mice (Vital River, China) were female. BLAB/c mice aged 6 weeks with a body weight of 15–17 g and *MT*−/− and *MT*+/+ mice aged 8 weeks with a body weight of 20–22 g were used. All the mice were monitored under specific pathogen-free conditions in the Department of Laboratory Animal Science of Peking University Health Science Center. The animals were housed in an air-conditioned animal room with a constant temperature of 23 ± 2°C and a relative humidity of 40 ± 5%. A standard diet and water were provided by the laboratory. All the studies were approved by the Biomedical Ethics Committee of Peking University, and all the methods were performed in accordance with relevant guidelines and regulations.

### Establishment of the DNFB-induced AD-like murine model

The DNFB-induced AD-like murine model was developed using a standard procedure^18^. Briefly, the 2.4-dinitrofluorobenzene (DNFB) (Wako, Japan) was diluted in a mixture of acetone and olive oil (4:1). Then, 100 *μ*L of 0.5% DNFB was applied to the shaved back of the mice (3 × 2 cm^2^) in the first week for sensitization. Subsequently, 100 *μ*L of 0.2% DNFB was applied twice a week for an additional 4 weeks to induce AD-like lesions. The model was established at the end of the fifth week. The role of MT on the DNFB-induced AD-like model in BALB/c mice was assessed from Day 1 to Day 14 after the model was established.

### Group assignment

BALB/c mice were randomly divided into five groups (*n* = 5 for each group), including a control group and four model groups. The four model groups were sacrificed on Day 1, Day 3, Day 7, and Day 14, respectively, after the model was created. The control group mice were treated with a mixture of acetone and olive oil when the model groups were induced by DNFB, and the control mice were sacrificed at the end of fifth week. Dorsal skin from a fixed position on the mice was biopsied for MT mRNA and protein measurement, histopathology, and an immunohistology assessment.

There were five mice in both the *MT*−/− and *MT*+/+ groups. They were sacrificed on Day 1 after the creation of the DNFB-induced AD-like model at the end of fifth week. The peripheral whole blood was obtained by retro-orbital bleeding for flow cytometry to assess CD3, CD4, CD8, CD25, IL-4, IFN-γ, IL-17A, and FoxP3. Dorsal skin from a fixed position on the mice was biopsied. A quarter of the biopsy was lysed to extract the entire protein for ELISA. A quarter was fixed in formaldehyde for histopathology and another quarter for CD4 and TSLP immunostaining. The last quarter was lysed to extract total RNA for NDUFAF1 mRNA measurement, as described.

The following parameters were observed:

### Evaluation of the Skin Lesions

The dorsal skin of each group was photographed before and after the treatment. Skin lesions such as erythema, edema, exfoliation, and scaling were scored as 0 (none), 1 (mild), 2 (moderate), and 3 (severe) and assessed blind by two individual dermatologists^17,18^.

### Thickness of Skin

Dorsal skin thickness was measured before the mice were sacrificed using a spring micrometer (Hautine International Co., China) with a precision of 0.01 mm. For each mouse, three different sites of the dorsal skin were measured. Three repetitions were measured for each site, and an average of the data was calculated.

### Weight of Skin

A square of dorsal skin measuring 6 mm × 6 mm from a fixed position on each mouse was sheared without subcutaneous fat. The weight of skin was measured with an electronic balance (Sartorius CP225D, Germany).

### RNA Isolation and Quantitative Real-Time Polymerase Chain Reaction

Total RNA from the dorsal skin was extracted with an E.Z.N.A.® Tissue RNA Kit (Omega, USA). The RNA concentration was measured with an ultraviolet spectrophotometer (Thermo, Germany). According to the manufacturer’s protocol, purified total RNA (1 μg) was reverse transcribed into complementary DNA (cDNA) with the M-MLV first strand cDNA Synthesis Kit (Omega, USA) in a cycle of 60 minutes of incubation at 37 °C, followed by 5 minutes at 85 °C, and subsequent chilling on ice. After the cDNA samples were diluted 20 times with distilled water, each sample was mixed with primers (designed using NCBI), distilled water, and the SYBR Premix Ex Taq (TaKaRa Bio, Dalian) in a 20 μL reaction system. Quantitative real-time PCR was performed in an iQ5 real-time PCR system (Bio Rad, USA). The *MT-1* primers were as follows: forward 5′-ACCCCAACTGCTCCTGCT-3′ and reverse 5′-GCGCCTTTGCAGACACAG-3′, which provided a 154 bp PCR product^30^; and the *MT-2* primers were as follows: forward 5′-TCGACCCAATACTCTCCGCT-3′ and reverse 5′-GATCCATCGGAGGCACAGGA-3′, which provided a 149 bp PCR product. The expression of β-actin gene was used as an endogenous control to normalize the mRNA expression of *MT-1* and *MT-2*. The β-actin primers were as follows: forward 5′-GCTTCTTTGCAGCTCCTTCGT-3′ and reverse 5′-AGCGCAGCGATATCGTCATC-3′^17^, which provided a 73 bp PCR product. The NDUFAF1 primers were as follows: forward 5′-CTCTAATCGAGGAAGAATCCG-3′ and reverse 5′-AGAATGGACCATCCACTTTATC-3′, which provided a 101 bp PCR product (designed by NCBI). The ACTB gene was used as an endogenous control to normalize the mRNA expression of NDUFAF1. The ACTB primers were as follows: forward 5′-GGCTCCTAGCACCATGAAGA-3′ and reverse 5′-AGCTCAGTAACAGTCCGCC-3′, which provided a 187 bp PCR product. All the primers were synthesized by Sagon Bio (Shanghai, China). The PCR conditions were as follows: a hold time of 30 s at 95 °C, denaturation at 95 °C for 5 s, annealing at 60 °C for 30 s, and looping 40 times. The results of the CT values were transferred into 2^−ΔΔCT^.

### Enzyme-Linked Immunosorbent Assay (ELISA)

Dorsal skin in an area measuring 6 mm × 6 mm from a fixed position was sheared without subcutaneous fat. Levels of *MT-1* (Fankewei Bio, China), IL-4, IL-5, IL-6, IL-10 (ebioscience, China), malondialdehyde (MDA), nitric oxide synthases (NOS), and superoxide dismutase (SOD) (Nanjing, China) in the *MT*−/− and *MT*+/+ skin tissue homogenate supernatant were measured using ELISA kits, following the manufacturer’s instructions.

### Immunohistology

The primary antibody for MT (AB192385, 1:50, no heat-mediated antigen retrieval), CD4 (AB183685, 1:1000, heat-mediated antigen retrieval with Tris/EDTA buffer at pH 9.0), and TSLP (AB115700, 1:200, perform heat-mediated antigen retrieval with a citrate buffer at pH 6.0) were purchased from Abcam Company (UK). The secondary antibodies (PV-6001) and DAB stain were all purchased from Zhongshan Golden Bridge Biotechnology (Beijing, China). Images of each section were obtained under the microscope (Nikon E600). The staining intensity without counterstaining for MT was scored on a four-point scale, as follows: 0, no positive staining; 1+, mild cytoplasmic staining; 2+, moderate-to-severe cytoplasmic staining; and 3+, moderate-to-severe cytoplasmic staining with nuclear staining^31^.

Average CD4+ T cell numbers in the skin were analyzed by measuring five different continuous areas in each skin section (×200) (Image-Pro Plus 6.0.0.260, Media Cybemetics).

### Flow cytometry

The peripheral blood of the *MT*−/− and *MT*+/+ mice was obtained by retro-orbital bleeding and was heparinized immediately to obtain peripheral whole blood. For analysis of the expression of intracellular IL-4, IL-17A, IFN-γ, and FoxP3, single peripheral blood cell suspensions were prepared and stimulated with phorbol 12-myristate 13-acetate (PMA) and ionomycin (Sigma) for 4 hours at 37 °C with final concentrations at 1 μg/mL and 50 μg/mL, separately. GolgiPlug (BD Biosciences, San Diego, CA, USA) with a final concentration at 2 µM was also added into the culture to block intracellular cytokine transport processes. Cells were incubated with anti-mouse CD16/32 (BD Bioscience) at 4 °C for 5 minutes to block the Fc receptor. The cells were then incubated with APC-H7 for CD3, BUV395 for CD4, Percp-Cy5.5 for CD8, and PE-CY7 for CD25 (all the markers were purchased from BD Biosciences, USA) for 15 minutes at room temperature. After the cell surface marker staining, the cells were fixed and permeabilized using BD Cytofix/Cytoperm (BD Biosciences, USA). During permeabilization, the cells were incubated with PE-IL-4, FITC-IFN-γ, PE-CF 594-IL-17A, and AF 647-FoxP3 (BD Biosciences) for 15 minutes at room temperature. FACS was performed on a BD FACSAria II Special Order System (BD Bioscience).

### Statistical analysis

The statistical analysis was performed with SPSS 16.0. All the data that followed a normal distribution were analyzed using the t-test, and data that followed non-normal distribution were assessed using a nonparametric test. A *P* value less than 0.0125 in the BLAB/c mice groups and 0.05 in the *MT*−/− vs. *MT*+/+ group was considered significant.
